# Bioabsorbable metal zinc differentially affects mitochondria in vascular endothelial and smooth muscle cells

**DOI:** 10.1016/j.bbiosy.2021.100027

**Published:** 2021-08-26

**Authors:** Olivia R.M. Bagshaw, Fereshteh Moradi, Christopher S. Moffatt, Hillary A. Hettwer, Ping Liang, Jeremy Goldman, Jaroslaw W. Drelich, Jeffrey A. Stuart

**Affiliations:** a Department of Biological Sciences, Brock University, 1812 Sir Isaac Brock Way, St. Catharines, Ontario L2S3A1, Canada; b Department of Biomedical Engineering, Michigan Technological University, 1400 Townsend Drive, Houghton, MI 49931, United States; c Department of Materials Science and Engineering, Michigan Technological University, 1400 Townsend Drive, Houghton, MI 49931, United States

**Keywords:** Zinc, Endothelial cell, Smooth muscle cell, Biodegradable, Stent

## Abstract

Zinc is an essential trace element having various structural, catalytic and regulatory interactions with an estimated 3000 proteins. Zinc has drawn recent attention for its use, both as pure metal and alloyed, in arterial stents due to its biodegradability, biocompatibility, and low corrosion rates. Previous studies have demonstrated that zinc metal implants prevent the development of neointimal hyperplasia, which is a common cause of restenosis following coronary intervention. This suppression appears to be smooth muscle cell-specific, as reendothelization of the neointima is not inhibited. To better understand the basis of zinc's differential effects on rat aortic smooth muscle (RASMC) versus endothelial (RAENDO) cells, we conducted a transcriptomic analysis of both cell types following one-week continuous treatment with 5 µM or 50 µM zinc. This analysis indicated that genes whose protein products regulate mitochondrial functions, including oxidative phosphorylation and fusion/fission, are differentially affected by zinc in the two cell types. To better understand this, we performed Seahorse metabolic flux assays and quantitative imaging of mitochondrial networks in both cell types. Zinc treatment differently affected energy metabolism and mitochondrial structure/function in the two cell types. For example, both basal and maximal oxygen consumption rates were increased by zinc in RASMC but not in RAENDO. Zinc treatment increased apparent mitochondrial fusion in RASMC cells but increased mitochondrial fission in RAENDO cells. These results provide some insight into the mechanisms by which zinc treatment differently affects the two cell types and this information is important for understanding the role of zinc treatment in vascular cells and improving its use in biodegradable metal implants.

## Highlights

• Zinc-based models of aortic stents differentially affect rat aortic smooth muscle and endothelial cells *in vivo*.
• Seven-day exposure to 5 µM or 50 µM ZnSO_4_ affects several key nuclear-encoded mitochondrial genes in smooth muscle and endothelial cells.
• Concomitantly, 5 µM or 50 µM ZnSO_4_ affects cellular energy metabolism and mitochondrial network dynamics, but differently in both cell types.
• Zinc affects mitochondrial form and function differently in smooth muscle and endothelia cells, which may underly *in vivo* observations.

## Introduction

Percutaneous coronary intervention is a common practice to establish revascularization during stenotic arterial disease. This procedure is typically accompanied by the surgical placement of a stent to prevent post-angioplasty restenosis [1]. In the past, such stents remained in the artery for the lifetime of the patient resulting in the development of adverse side effects including smooth muscle neointimal hyperproliferation, thrombogenesis and destructive inflammation [2,3]. The advent of biodegradable/ bioabsorbable metal stents, which persist long enough to allow the return of normal vascular function, has been useful in preventing the development of long-term side effects.

Several metals have been evaluated for use in biodegradable/bioabsorbable stents including iron and magnesium, both as pure metal and alloyed. However, assessment of these metals has revealed several limitations; iron has a low corrosion rate- leading to similar adverse effects as found in permanent stents, and magnesium has poor mechanical strength and increased corrosion rates leading to local alkalization and early loss of mechanical integrity [4], [5], [6], [7], [8], [9], [10], [11]. More recently, there has been significant progress in the use of zinc-based alloys for bioabsorbable stents. Zinc is an essential trace metal in the body which interacts with an estimated 3000 proteins; participating in several critical cellular functions including nucleic acid/protein metabolism as well as cell growth and division [12], [13], [14]. Recent studies have observed a beneficial suppressive effect of neointimal hyperplasia in the presence of zinc metal implants. In a model of stent implantation, a zinc metal wire was surgically placed in the abdominal aorta of Sprague-Dawley rats for 6 months. Here, confluent endothelization along the luminal surface of the zinc metal wire implant was observed in addition to a decreasing gradient of smooth muscle cells close to the implant surface [15]. Consistent with this, a similar gradient was observed with pure zinc stent implants in rabbit abdominal aorta [16]. These results suggest that zinc corrosion products released from metal implants suppress smooth muscle cell hyperproliferation but do not affect endothelization or smooth muscle cells in the medial layer of the aorta [15,17]. To examine this effect further, an ex vivo arterial culture study washed tissue slices prepared from the thoracic aorta of rats with 0.5–1.5 mM Zn-acetate to simulate zinc corrosion products. Suppression of caspase-8 and -9 activity was observed at high concentrations, as well as a dose-dependent activation of caspase-3 activity [17]. These results are consistent with studies that have demonstrated decreased cell viability in response to zinc exposure [6,10]. While it may be accepted that zinc acts as a potent inhibitor of caspase-3,-8 and -9 activity at concentrations above 100 µM, most of such studies have been performed in isolated enzyme systems which lack many of the critical compartments, organelles and competing proteins which may render such effects negligible *in vivo* [17], [18], [19], [20]. Collectively, these results indicate a complex relationship between zinc exposure and caspase activation in intact cells which likely translates to other zinc effects evaluated in isolated mitochondria.

We describe herein the effects of one-week continuous zinc exposure in vascular cells to model their response to zinc-based stents *in vivo*. Previous studies have evaluated the cellular response of rat aortic smooth muscle and endothelial cells for up to 24 h [6,10]. While this approach may highlight cellular signaling pathways targeted by exogenous zinc, it does not model the more relevant physiological adaptation of cells exposed to a slowly degrading metal implant *in vivo*. A typical zinc-based stent is approximately 50 mg of pure metal [22], [23], [24]. If we assume the stent degrades completely within 18 months (ideal bioabsorption: 12–24 months), this results in approximately 90 µg zinc released per day on average [22,25]. High velocity blood flow in coronary arteries (∼250 ml/min) likely facilitates rapid clearance of zinc degradation products from the lumen [26], but not those released from the surface in contact with the neointima surrounding the stent [17]. We do not have data on the concentrations of zinc that develop in the neointima, and indeed a concentration gradient will be present from the stent to surrounding tissue cells. We chose to treat cells with a relatively low concentration of zinc (5 µM) and a ten-fold higher concentration (50 µM). We predict that, *in vivo*, cells in the neointima of a stent-containing artery would encounter zinc concentrations within this range.

RNA-seq analyses of rat aortic endothelial and smooth muscle cells in these experiments revealed significant effects of zinc on gene expression in both cell types, with a primarily mitochondrial response at low concentration (5 µM) and upregulation of proteins required for regulation of metal homeostasis at higher concentrations (50 µM). Evaluation of cellular respiration and mitochondrial network characteristics of both cell types following zinc treatment revealed differential effects on oxidative phosphorylation and mitochondrial dynamics.

## Experimental section

### Zn^2+^ solution

ZnSO_4_-heptahydrate (Sigma, Z-0251) was dissolved in Milli-Q water from a GenPure UV/UF xCAD plus Ultrapure water purification system (Thermo Scientific, 50136151) and subsequently autoclaved. 100 mM stock solutions were stored at -20 °C and serial dilutions were made in sterile-filtered (0.20 µM polyethersulfone) Milli-Q water prior to each experiment.

### Cell culture

Primary rat aortic endothelial cells (Cell Biologics, RN-6052) and rat aortic smooth muscle cells (Lonza, R-ASM-580) were cultured in physiologically representative media (Plasmax) formulated as described in [21]. Plasmax media was supplemented with 10% fetal bovine serum (Sigma, F1051), 1% penicillin/streptomycin (Sigma, P4333), 6.5 mM L-glutamine and 1 mM sodium pyruvate to ensure adequate growth of primary cells. Cells were incubated in physiologically relevant oxygen levels within a 5% CO_2_/ 5% O_2_ humidified incubator (Keeley and Mann 2019). Sub-culture was performed with 0.25% tryspin/EDTA solution (Sigma, T4049). Media was refreshed every 24 h. Cells were cultured for 7 days at a final ZnSO_4_ concentration of 0 µM (Control), 5 µM, 50 µM.

### RNA isolation and sequencing

Total cellular RNA was extracted isolated using the RNEasy Plus Mini Kit (Qiagen, 74131) according to the manufacturer's protocol. RNA concentration and purity were evaluated as A260/280 ratio using a Thermo Fisher Scientific Nanodrop spectrometer. RNA samples were then snap frozen in liquid nitrogen and stored at -80 °C until being sent to Novogene (Sacramento, CA) for sequencing and analysis. Pair-ends at 150 bp (PE150) high throughput Illumina sequencing was performed at read-depth of 40 million reads. The HISAT2 algorithm [27] was used to align reads to the reference genome (*Rattus Norvegicus*). Gene expression levels were estimated by calculating FPKM (fragments per kilobase of transcript per million mapped sequence reads), which were further adjusted using trimmed mean of M values (TMM) [28] before differential expression analysis was performed using the EdgeR *R* package [29].

### Gene ontology (GO) term analysis

GO term analysis was performed using the Database for Annotation, Visualization and Integrated Discovery (DAVID) v.6.8 with the default settings (https://david.ncifcrf.gov) [30,31]. The GO direct annotation level was used to obtain enriched GO terms. Gene lists of differentially expressed genes (DEG) provided by Novogene were reduced to genes with a Benjamini adjusted *p*-value < 0.005 in order to produce a concise list of GO terms which reflect the most strongly affected genes.

### Cellular respiration

Cellular respiration parameters were measured using a Seahorse Extracellular Flux Analyzer XF^e^24 Mito Stress test (Agilent, Santa Clara, CA). Measurements were performed using Plasmax media without serum, sodium bicarbonate or penicillin/streptomycin supplementation. The Mito Stress Test was performed using 1 µM oligomycin A, 1 µM FCCP and 0.5 µM Rotenone/antimycin A, added according to standard protocol. At the end of the test, cells were trypsinized, isolated and counted in triplicate using a hemocytometer. Oxygen consumption rate (OCR) and extracellular acidification rate (ECAR) were normalized to total cells in all experiments.

### Fluorescence microscopy

Cells were seeded on 35 mm Mattek cell culture imaging plates (L-lysine-coated) on Day 5 of zinc treatments. On Day 7, media was removed and replaced with media containing 10 nM tetramethyl rhodamine methyl ester (TMRM) (Biotium, 70017) and incubated for 30 min. Images were obtained on a Zeiss Cell Observer SD spinning disk confocal microscope equipped with Teledyne Photometric Prime BSI and Yokogawa Sensor Cameras. Images were analyzed using ImageJ.

### Measurement of mitochondrial membrane potential

TMRM fluorescence images were cropped into individual cells, while minimizing overlap of nearby cells. Cells containing multiple nuclei (actively dividing) or which were too close together to reliably delineate individual cells were not used in the analysis. TMRM is a lipophilic cation that accumulates in the mitochondria according to the mitochondrial membrane potential. TMRM fluorescence was analyzed using our own TMRM analysis script for the Fiji distribution of ImageJ. This tool uses a thresholding operation (Otsu) to designate all pixels as being either mitochondrial or background [32]. The average pixel intensity is then calculated for both mitochondria and background, and the average background intensity is subtracted from the average mitochondrial intensity for each image.

### Quantitative analysis of mitochondrial network morphology

Mitochondrial network morphology was assessed using the Mitochondrial Network Analysis Tool (MiNA) for the Fiji distribution of ImageJ [33]. Several pre-processing tools were used to enhance contrast and sharpen mitochondrial images, to ensure quantification by MiNA that closely represents the true fluorescence image. Differences in mitochondrial network abundance and complexity may require different image pre-processing to ensure that MiNA detection is accurate. For RASMC images, an unsharp mask (sigma=1) was used to sharpen edges by subtracting a blurred version of the image (unsharp mask) from the image. To create the unsharp mask, the original image undergoes Gaussian blurring which is then multiplied by the mask weight (0.8). Second, a median filter (radius=1) was applied to enhance contrast by replacing each pixel with the neighbourhood median. For RAENDO images, an unsharp mask (sigma=3) and a mask weight (0.9) were used followed by a median filter (radius=1). Briefly, images underwent thresholding using the Otsu thresholding operation to produce a binary image [32]. Mitochondrial footprint was calculated from the total area of mitochondrial-signal positive pixels. Mitochondrial form interconnected, branching networks in which branches are connected at a node. Following binarization, a morphological skeleton was then produced using the Skeletonize 2D/3D plug-in [34,35]. This method employs iterative thinning to create a skeleton of mitochondrial structures, one pixel wide. Length measurements are then deduced from the morphological skeleton using the Analyze Skeleton plug-in. Mean branch length is calculated as the average length of a mitochondrial structure between two nodes. Mean network size was calculated by computing the sum of all branch lengths within an independent network and dividing this by the total number of individual networks within a cell.

### Statistical analysis

Data are presented as means ± standard deviation. A one-way analysis of variance (ANOVA) with Tukey's multiple comparisons post-hoc analyses were performed for each data set. *P* values ≤ 0.05 were considered significant. All statistical analyses were performed using GraphPad Prism version 9.1.0 (San Diego, CA).

## Results

### 5 µM Zn^2+^ treatment targets primarily mitochondrial transcripts while 50 µM induces primarily metal regulatory pathways

In initial experiments we measured the effects of continuous ZnSO_4_ treatment for 7 days on the growth and viability of RAENDO and RASMC cells (Fig. S1). At concentrations up to 50 µM, ZnSO_4_ did not affect either growth or viability. We elected to use 5 µM and 50 µM as representative sublethal low and high ZnSO_4_ treatments.

**Fig. 1:**
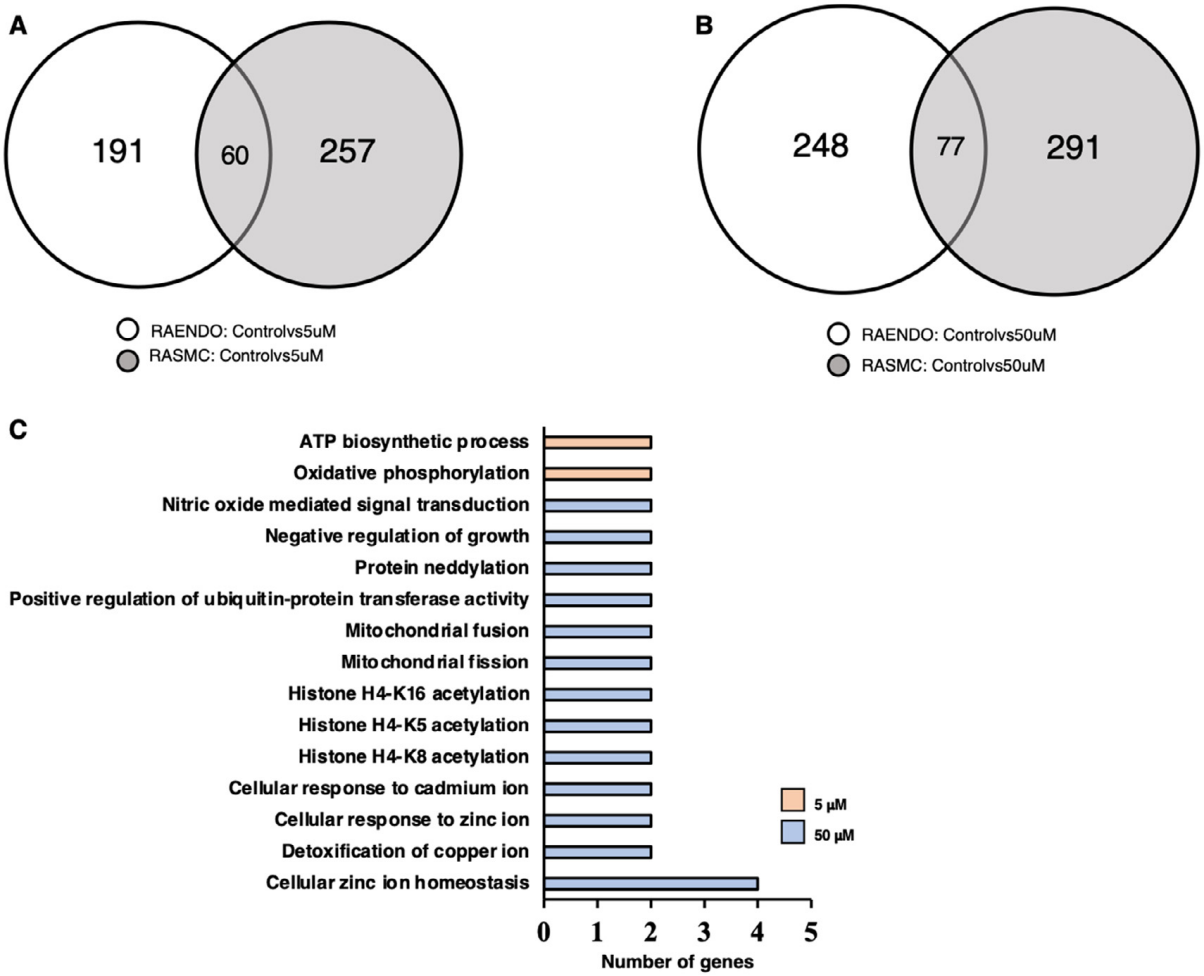
Venn diagrams of differentially expressed genes (DEGs) in RAENDO and RASMC cells treated with (a) 5 µM ZnSO_4_ or (b) 50 µM ZnSO_4_ compared to control (0 µM). (c) GO terms (biological process) of DEGs associated with the overlapping regions of Venn diagrams.

To evaluate the effect of long-term zinc supplementation on rat aortic endothelial and smooth muscle global gene expression, we treated cells with 0 (Control), 5 µM or 50 µM of ZnSO_4_ for 7 days, following which RNA was extracted and subsequently sequenced by Novogene (Sacramento, CA). 5 µM ZnSO_4_ treatment elicited differential expression of 251 transcripts in endothelial cells and 317 in smooth muscle cells. 60 of these genes were differentially regulated in both endothelial and smooth muscle cells (Fig. 1). GO term analysis using the DAVID tool indicated that these 60 genes were associated primarily with ATP biosynthetic processes and oxidative phosphorylation, suggesting a mitochondrial and/or metabolic response at this concentration (Fig. 1A,C). 50 µM ZnSO_4_ treatment elicited differential expression of 325 genes in endothelial cells and 368 genes in smooth muscle cells. Of these, 77 genes were commonly differentially regulated in both endothelial and smooth muscle cells. GO term analyses indicated that these 77 genes were associated with several biological processes including mitochondrial fusion/fission dynamics, histone acetylation, negative regulation of growth, nitric oxide signalling, post-translational modification (i.e. neddylation, ubiquitination) and cellular response to/detoxification of metal ions (Fig. 1B,C).

**Fig. 2:**
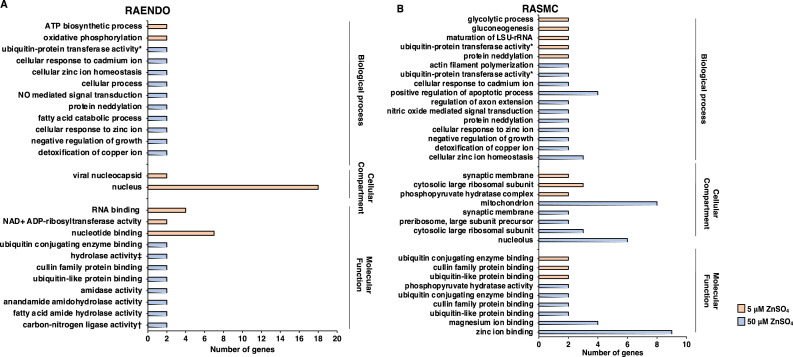
GO term analysis of differentially expressed genes in (a) RAENDO and (b) RASMC cells treated with 5 µM or 50 µM ZnSO_4_ compared to control (0 µM). DEG lists for this analysis were reduced to genes with the Benjamini adjusted *p*-value <0.005. *positive regulation of, †with glutamine as amido-N-donor, ‡acting on ester bonds.

GO term analyses were also performed on DEG lists comparing 5 µM and 50 µM treatments to the respective cell-type control. The DEG lists were reduced to genes which had a *p*-value <0.005 in order to produce very concise GO term lists. GO term analyses of gene lists containing genes with a *p*-value of <0.05 results in a very similar GO term enrichment profile with some terms being excluded when the *p*-value is reduced to <0.005 (data not shown). Several cell-type specific responses to chronic zinc treatment were observed (Fig. 2). At 5 µM ZnSO_4_, ATP biosynthetic processes and oxidative phosphorylation are strongly affected biological processes in RAENDO cells, whereas in RASMC cells glycolytic process and gluconeogenesis are affected. At 50 µM, both cell types demonstrate a strong effect of zinc treatment on metal ion regulation and cellular response to metal ions as well as protein neddylation and negative regulation of growth. The most strongly affected cellular compartment at 5 µM ZnSO_4_ in RAENDO cells was the nucleus, whereas in RASMC cells it was the cytosolic large ribosomal subunit. There are no enriched cellular compartment GO terms for RAENDO cells at 50 µM, but the mitochondrion and nucleolus are the most strongly affected in RASMC cells. At 5 µM, nucleotide binding is the most affected molecular function in RAENDO cells, whereas in RASMC cells various protein binding molecular functions are affected. At 50 µM, protein binding activity is affected in both cell types, with various hydrolase activities targeted in RAENDO cells and metal ion binding activity in RASMC cells. Notably, the most strongly affected molecular function for RASMC cells treated with 50 µM ZnSO_4_ was zinc ion binding.

**Fig.3:**
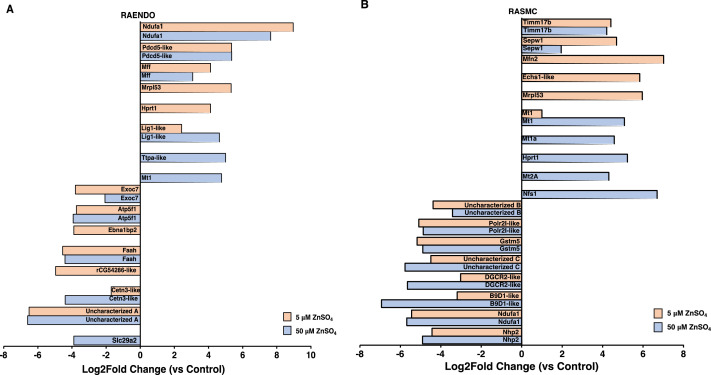
Significantly differentially expressed genes in (a) RAENDO and (b) RASMC cells treated with 5 µM or 50 ZnSO_4_ treatment compared to control (0 µM). The top 5 most significant protein-coding DEGs were selected from each gene list, and compared within cell types. Ndufa1; NADH dehydrogenase (ubiquinone) 1 α subcomplex 1, Pcdp5-like; programmed cell death protein 5-like (ENSEMBL gene ID: ENSRNOG00000050827), Mff; mitochondrial fission factor, Mrpl53; mitochondrial ribosomal protein L53, Hprt1; hypoxanthine phosphoribosyltransferase 1, Lig1-like; DNA ligase 1-like (ENSEMBL gene ID: ENSRNOG00000047206), ttpa-like; tissue-type plasminogen activator like (ENSEMBL gene ID: ENSRNOG00000060898), Mt1; metallothionein 1, Exoc7; exocyst complex component 7, Atp5f1; ATP synthase H+ transporting mitochondrial Fo complex subunit B1, Ebna1np2; EBNA1 binding protein 2, Faah; fatty acid amide hydrolase, rCG54286-like; LOC1003619 (ENSEMBL gene ID: ENSRNOG00000020628), Cetn3-like; centrin-3-like (ENSEMBL gene ID: ENSRNOG00000050877), Uncharacterized A; AABR07066510.1 (ENSEMBL gene ID: ENSRNOG00000049123), Slc29a2; solute carrier family 29 member 2, Timm17b; translocase of the inner membrane 17b, Sepw1; selenoprotein W1, Mfn2; mitofusin 2, Echs-like; enoyl-CoA hydratase mitochondrial-like (ENSEMBL gene ID: ENSRNOG00000047565), Mt1a; metallothionein 1A, Mt2a; metallothionein 2A, Nfs1; NFS1 cysteine desulferase, Uncharacterized B; LOC100909555 (ENSEMBL gene ID: ENSRNOG00000047746), Polr2l-like; DNA-directed RNA polymerase II subunit RPB9-like, Gstm5; glutathionine S-transferase mu 5, Uncharacterized C; AABR07028989.1 (ENSEMBL gene ID: ENSRNOG00000050441, DGCR1-like; integral membrane protein DGCR2/IDD-like (ENSEMBL gene ID: ENSRNOG00000049965), B9D1-like; B9 domain containing protein 1-like (ENSEMBL gene ID: ENSRNOG00000002462), Nhp2; NHP2 ribonucleoprotein.

Genes related to mitochondrial processes, including oxidative phosphorylation (e.g., *Ndufa1* and *Atp5f1*), protein import (*Timm17b*) and network dynamics (*Mff* and *Mfn2*) were among the transcripts with the greatest differences in levels in response to ZnSO_4_ (Fig. 3). In addition, several metallothionein genes are up-regulated in response to zinc treatment with no concomitant differential regulation of zinc-specific transporter proteins (e.g., ZnT or ZIP). Notably, the complex 1 gene *Ndufa1* was up-regulated in RAENDO cells, but down-regulated in RASMC cells. Transcripts related to mitochondrial network dynamics also showed different responses in the two cell types. Up-regulation of mitochondrial fission factor (*Mff*) in RAENDO cells suggests increased mitochondrial network fission, whereas up-regulation of mitofusin-2 (*Mfn2*) in RASMC cells suggests increased mitochondrial fusion. Together these data indicate that mitochondria are a major target of ZnSO_4_, and we therefore focused on analyses of mitochondrial form and function in cells exposed to ZnSO_4_ continuously for 7 days.

### Chronic zinc treatment has no effect on RAENDO OCR or ECAR but stimulate RASMC OCR and ECAR at 50 µM

**Fig.4:**
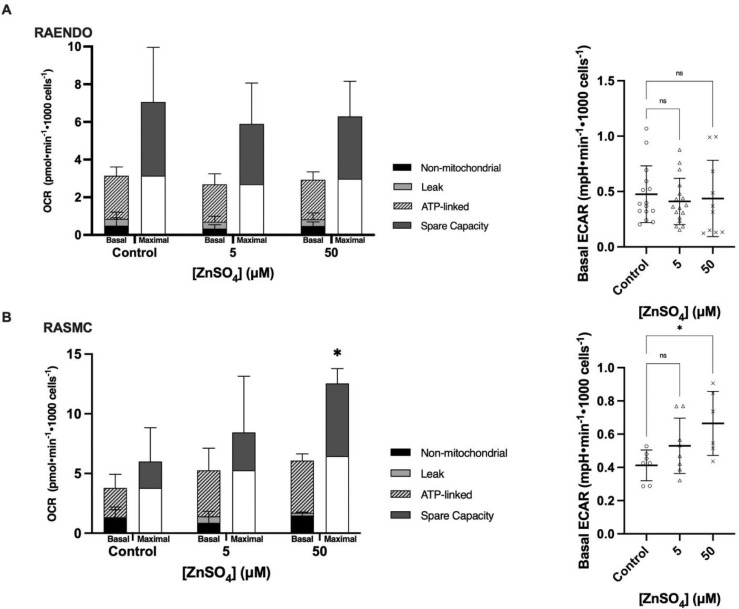
Basal and maximal oxygen consumption rates (OCR) and extracellular acidification rates (ECAR) of (a) RAENDO and (b) RASMC following 7 days of treatment with 0–50 µM ZnSO_4_. Basal oxygen consumption is subdivided into non-mitochondrial (Non-mitochondrial), proton leak oxygen consumption (Leak) and ATP-linked oxygen consumption (ATP-linked). Spare respiratory capacity (Spare Capacity) is indicated on the maximal OCR bar. * *p*-value ≤ 0.05. *n* = 4–12.

To determine the effect of chronic zinc supplementation on cellular energy metabolism, a Seahorse Mito Stress Test was performed following 7 days of treatment. No significant changes in basal or maximal OCR were observed in RAENDO cells at either concentration of ZnSO_4_ (Fig. 4a). Similarly, there were no effects on ATP-linked or proton leak-linked OCR in these cells, or on ECAR, suggesting that zinc supplementation has little significant effect on the energy metabolism of RAENDO cells.

In contrast, ZnSO_4_ treatment had substantial effects on energy metabolism in RASMC cells. Dose-dependent increases in basal and maximal OCR were observed concomitantly with dose-dependent increases in ECAR (Fig. 4B).

### Chronic zinc treatment reduces mitochondrial membrane potential in a dose-dependent manner in both cell types but does not affect apparent mitochondrial abundance

**Fig. 5:**
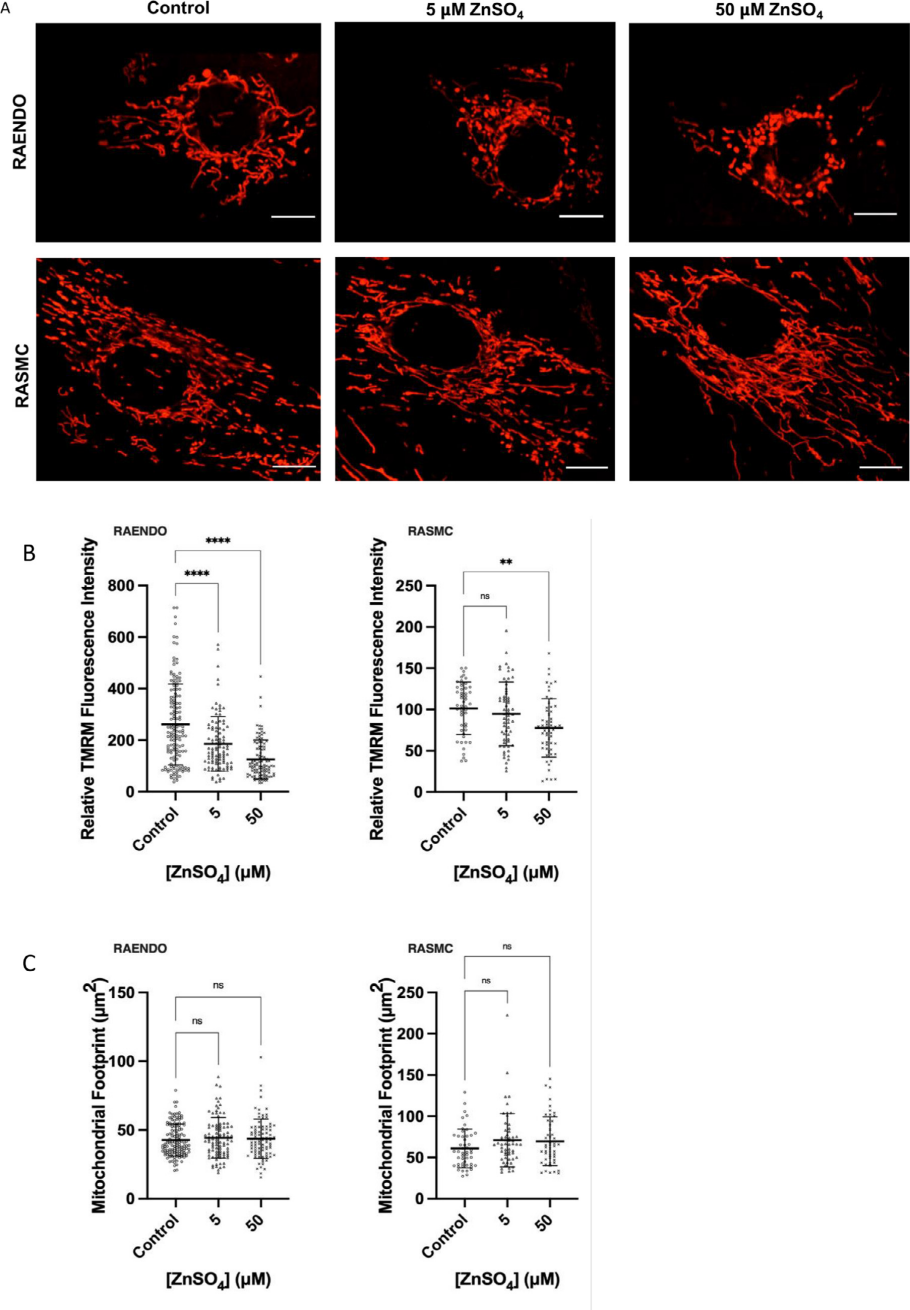

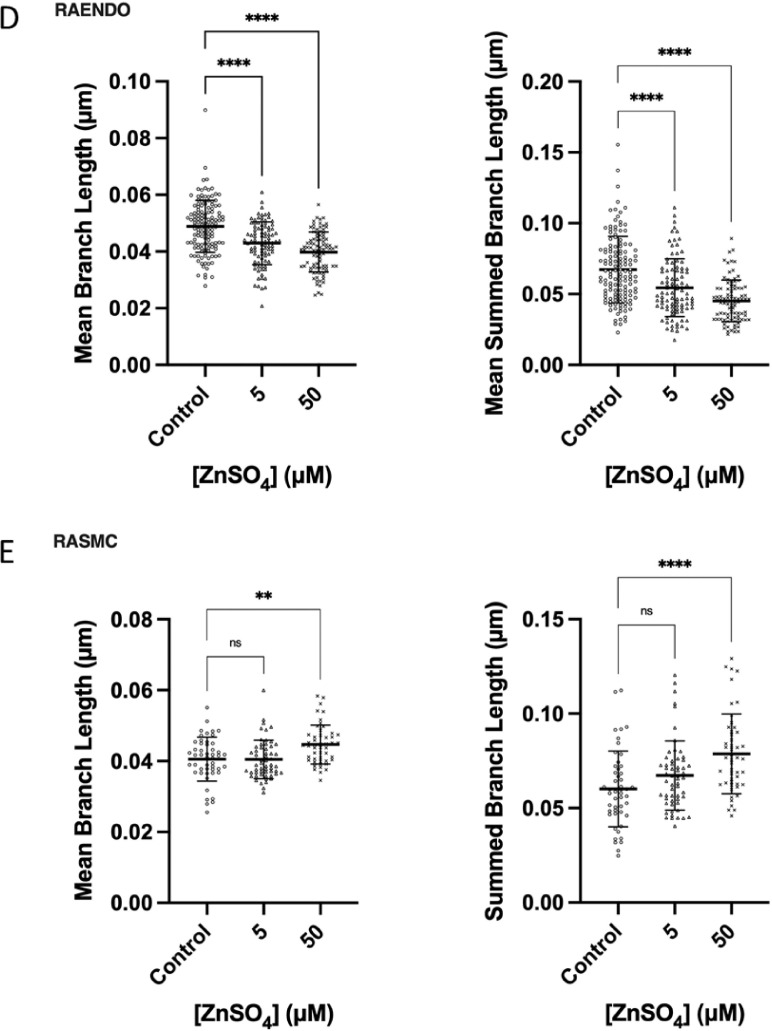
Effects of 7 days continuous treatment with ZnSO_4_ on mitochondrial parameters in live TMRM-stained cells. (a) Live cell TMRM fluorescence images of RAENDO and RASMC cells treated with 0–50 µM ZnSO_4_. On Day 7, cells were incubated with 10 nM TMRM for 30 min and subsequently imaged at 630x magnification using a Zeiss Cell Observer SD spinning disk confocal microscope. Scale bars represent 10 µm. (b,c) Relative mitochondrial membrane potential and mitochondrial footprint of (b) RAENDO and (c) RASMC. Cells were seeded on Day 5 of treatment. On Day 7, media was replaced and 10 nM TMRM added. Relative fluorescence intensity was evaluated to determine relative mitochondrial membrane potential. Total area of mitochondrial signal positive pixels was evaluated to determine mitochondrial footprint. (d–e) Mitochondrial network morphology analysis (MiNA) results for (d) RAENDO and (e) RASMC cells. Mean branch length was defined as the average length of a mitochondrial structure between two nodes. Mean network size was calculated by computing the sum of all branch lengths within an independent network and dividing this by the total number of individual networks within a cell. For b-e, ** *p*-value ≤ 0.01, **** *p*- value ≤ 0.0001. *n* = 49–144.

We qualitatively determined mitochondrial membrane potential using TMRM, a positively charged fluorescent dye that accumulates in the mitochondrial matrix relative to the membrane potential (Fig. 5).

Interestingly, ZnSO_4_ treatment induced declines in membrane potential in both cell types, despite the absence of metabolic effects in RAENDO cells (Fig. 5B). Mitochondrial footprint (Fig. 5C) is the area of the cell in the binarized TMRM image that is occupied by mitochondria. This is a proxy of mitochondrial abundance, where an increase or decrease may indicate mitochondrial biogenesis or mitophagy, respectively. Interestingly, there was no evidence that mitochondrial footprint was affected by ZnSO_4_ treatment in either cell type.

### Chronic zinc treatment cell-type specifically affects network morphological parameters

Mitochondria form dynamic, branching networks in which individual mitochondrial structures can fuse with or divide from a larger mitochondrial network. These processes are highly regulated and responsive to changes in activities of two of the gene products identified as being differentially expressed in the RNA-seq experiment: *Mff* and *Mfn2*. To evaluate mitochondrial morphology, we used our mitochondrial network analysis (MiNA) tool for the Fiji distribution of ImageJ. We measured mean branch length and mean summed branch length. Mean branch length and mean summed branch length measure the average length of mitochondrial structures, and the average summed length of branches within a network respectively, allowing for quantitative assessment of the extent to which mitochondria are fused into larger structures or fragmented into smaller structures. In RAENDO cells, a dose-dependent reduction in mean branch length (Fig. 5D) and mean summed branch length (Fig. 5E) was observed, consistent with greater mitochondrial fragmentation. This result is in agreement with the dose-dependent reduction in mitochondrial membrane potential and increased expression of *Mff* [36,37]. In RASMC cells, a dose-dependent increase in mean branch length and mean summed branch length, which suggests that chronic zinc treatment enhances mitochondrial fusion and/or inhibits mitochondrial fission. A dose-dependent reduction in mitochondrial membrane potential is also observed in RASMC cells, which in addition to larger network structures, may indicate a mechanism of mitochondrial complementation to optimize mitochondrial function during wide-spread mitochondrial dysfunction or enhanced energy demands [37,38]. These results are consistent with our RNA-seq data, where RASMC demonstrate an up-regulation of the mitochondrial fusion gene, mitofusin-2 (*Mfn2*).

## Discussion

Materials selected for use as stents are ideally biologically inert so as to not interfere with local or systemic biological function. However, materials used in the past have failed to prevent the development of neointimal hyperplasia when applied in an atherosclerotic artery, leading to incorporation of drug-eluting coatings that slowly release drugs such as rapamycin to prevent the development of smooth muscle hyperplasia [17,39,40]. Several studies have indicated that aortic zinc metal implants result in confluent endothelization along the neointima and a decreasing gradient of smooth muscle cells near the implant surface [15], [16], [17]. These results suggest that zinc-based stents may function as a possible alternative to drug-eluting stents for the prevention of in-stent restenosis *in vivo*.

Guillory et al. hypothesized that corrosion products released from zinc implants exerts suppressive effects on smooth muscle cells via caspase-mediated apoptosis [17]. While it is well established that zinc plays a role in the determination and progression of apoptosis, the associated mechanisms and pathways are varied, cell-specific, and poorly understood [41]. Zinc has been reported to induce apoptosis through various pathways in several cell-types while demonstrating anti-apoptotic effects in others [12,[41], [42], [43], [44]]. Previous cell culture studies with aortic smooth muscle and endothelial cells have demonstrated a reduction in cell viability, proliferation and migration rate at concentrations at or above 100 µM [6,10]. Consistent with this, we saw no adverse effect of 5–50 µM ZnSO_4_ on either cell growth or viability over 7 days, while 100 µM ZnSO_4_ slowed growth modestly in both cell lines (Supplementary Fig. 1). In contrast, treatment of various cell-types with 50–100 µM ZnSO_4_ can enhance proliferative signalling and cell motility [45,46].

In most mammalian cells, intracellular zinc concentrations range from 100 to 500 µM, with the vast majority of zinc ions being tightly bound to proteins; thus the total ‘free’ zinc concentration is in the picomolar range [47,48]. In the cell, maintenance of zinc ion concentrations is essential to prevent interference with other metal ions. If concentrations exceed their normal range they will begin to bind where they would not otherwise under physiological conditions [49,50]. According to the Irving-Williams series in inorganic chemistry, zinc ions bind much more strongly to proteins than most other biologically relevant ions including iron, manganese, magnesium and calcium [50,51]. Consequently, zinc regulatory proteins have binding affinities which reflect these physiological ranges and zinc concentrations are strictly regulated under physiological conditions [48,52].

There are two main mechanisms by which zinc homeostasis is maintained; zinc transporting proteins such as zinc-transporters (ZnTs) and Zrt-Irt-like proteins (ZIPs) and the zinc buffer-storage system [53,54]. Recent studies using fluorescent zinc reporters indicate that mitochondria, ER, and Golgi act as potential sites of zinc buffers and storage in the maintenance of zinc homeostasis [55]. As a consequence, cell-type specific sensitivity to zinc supplementation may be partially determined by the size and activities of the aforementioned intracellular storage sites. Muscle cells characteristically have large amounts of mitochondria in order to adequately support the energetic demands of muscle contraction while endothelial cells tend to rely primarily on glycolysis in order to promote angiogenesis in hypoxic environments [56], [57], [58], [59]. Interestingly, our GO term analysis indicates differential gene expression related to primarily oxidative phosphorylation at 5 µM ZnSO_4_ in RAENDO cells compared to differential gene expression related to glycolysis and gluconeogenesis in RASMC cells.

Our RNA-seq data indicates that both RAENDO and RASMC cells up-regulate metallothionein (MT) genes in response to chronic zinc treatment but do not differentially regulate various zinc transporting proteins such as ZnTs/ZIPs. While ZnTs and ZIPs regulate the efflux and influx into the cytosol, respectively, MTs bind zinc to facilitate storage, buffering and transfer to other proteins [60,61]. Regulation of these proteins at the level of gene expression to maintain zinc homeostasis has been previously demonstrated with human coronary artery endothelial (HCAEC) and pulmonary artery smooth muscle cells (HPASMC) [62]. Abdo et al. indicated downregulation of *ZnT1, ZnT2* and *MT1* following treatment with 25 µM ZnSO_4_ for 2 h as well as up-regulation of *ZIP2* and *ZIP12* following zinc depletion with pyrithione in both HCAEC and HPASMC [62]. However, such changes in expression of ZnTs/ZIPs may represent an acute response to zinc supplementation and adaptation over several days may alter this response. Enhanced MT expression may represent the primary means of maintaining intracellular zinc homeostasis under chronic treatment. Interestingly, MT expression plays an important role in collateral flow recovery and angiogenesis which may provide additional benefits when stents are applied in diseased arteries [63].

Our data indicate that mitochondria are influenced by zinc supplementation in a cell-type specific manner. Mitochondrial form highly dynamic networks which undergo cycles of fusion and fission in response to various cellular stimuli. We demonstrate that zinc treatment of endothelial cells results in a dose-dependent increase in mitochondrial fission, whereas smooth muscle cells demonstrate a dose-dependent increase in mitochondrial fusion. Mitochondrial fission is regulated by several key players, including dynamin related protein 1 (DRP1) and mitochondrial fission factor (MFF) [36]. Consistent with morphology results, RNA-seq analyses showed an upregulation of *Mff* in endothelial cells at 5 µM and 50 µM ZnSO_4_. Mitochondrial fission is often indicative of mitochondrial damage or dysfunction as it typically precedes mitophagy, the autophagic elimination of mitochondria. In fact, depolarization of the mitochondrial membrane potential (MMP) enhances mitophagy by promoting ubiquitination of mitochondrial surface proteins and activation of mitochondrial fission machinery [37,64]. MFF recruits active and oligomeric DRP1 to the mitochondrial outer membrane to promote fission. A recent study demonstrated that DRP1 interacts with the mitochondrial zinc transporter, ZIP1 to focally reduce mitochondrial membrane potential by promoting zinc entry through the ZIP1-mitochondrial calcium uniporter (MCU) complex [65]. Following fission, dysfunctional mitochondria fail to recover membrane potential and are subsequently degraded by mitophagy [65]. Further, recent studies have determined that zinc entry into mitochondria through the MCU plays an important role in the development of mitochondrial dysfunction and promotion of cell death following ischemia [66]. Taken together, the relationship between mitochondrial fission machinery, MMP and metal ionic balance is complex and poorly understood but may play a role in the development of stably reduced MMP in cells treated with ZnSO_4_ over 7 days.

In smooth muscle cells, *Mfn2* is upregulated at 5 µM but not 50 µM, despite a dose-dependent increase in network morphological parameters indicative of fusion. MFN2 is a dynamin-like GTPase important for mitochondrial fusion, calcium homeostasis, and ER-mitochondrial contact [67]. Several studies have shown that MFN2 inhibits cell proliferation and cell-cycle progression in several cell-types. In fact, the MFN2 gene was originally implicated for its role in the suppression of vascular proliferative disorders such as smooth muscle hyperplasia [67], [68], [69]. Therefore, upregulation of this gene and/or activation of the MFN2 protein in the presence of zinc may slow cell-cycle progression and prevent restenosis *in vivo*. MFN2 is also important for establishing mitochondrial-ER contact sites [70]. Functional ER-mitochondrial coupling promotes efficient calcium uptake into mitochondria, resulting in higher mitochondrial membrane potential, oxygen consumption and ATP production [70], [71], [72]. During vascular injury, such as following angioplasty, vascular smooth muscle cells can undergo a phenotypic switch from a mature ‘contractile’ phenotype to a de-differentiated, highly proliferative and secretory phenotype, leading to the development of neointimal hyperplasia [73], [74], [75], [76], [77]. In addition, dysregulation of mitochondrial dynamics and/or mitochondrial-ER coupling can promote the secretory and proliferative phenotype of vascular smooth muscle cells [70,71,78]. Therefore, up-regulation of *Mfn2* during chronic zinc supplementation may promote mitochondrial-ER contact and mitochondrial calcium homeostasis, thus preventing hyperplasia and restenosis *in vivo*. It is unclear why *Mfn*2 is upregulated at 5 µM but not 50 µM ZnSO_4_, despite consistent enhanced mitochondrial fusion at this concentration. Mitochondrial fusion may also occur during periods of stress or increased ATP demand, as a means of maximizing mitochondrial function by mixing partially damaged components as a form of complementation [37,38]. Mitochondrial quality control can be maintained through the elimination of damaged proteins by proteases and refolding by chaperone proteins in the mitochondrial matrix [37,79,80]. Outer membrane proteins can be removed through the ubiquitin-proteosome pathway [37,81]. Our GO term analyses of differential gene expression indicate positive regulation of ubiquitin transferase activity and ubiquitin-related protein binding in both cell types. This may indicate an alternative mechanism for mitochondrial quality control during chronic zinc supplementation.

Several of our data suggest that treatment of smooth muscle cells with zinc leads to an increase in ATP demand. Enhanced basal and maximal oxygen consumption rates as well as extracellular acidification rates suggest that both mitochondrial and glycolytic ATP-producing reactions are working at faster rates. GO term analyses indicating differential regulation of genes associated with glycolysis and gluconeogenesis further indicate increased ATP demand. While previous studies have suggested that zinc acts as an inhibitor of cellular energy production both within the electron transport system and glycolysis, most of these studies were performed in cell-free systems in the absence of the cell's endogenous zinc-buffering capacity [82], [83], [84], [85], [86]. Increases in ATP demand may be relevant to increases in intracellular zinc concentrations, where zinc ions can disrupt binding of other essential metal ions such as magnesium. Magnesium homeostasis is essential for regulation of tricyclic acid cycle (TCA) enzymatic activity and maintenance of the electron transport system [87], [88], [89], [90]. Moreover, ATP binds to the magnesium ion to form the biologically functional form Mg-ATP. Mitochondrial ATP is transported from the mitochondria in the magnesium bound form by the ATP-Mg/Pi [87,[91], [92], [93]] Magnesium is also an essential co-factor for the sodium/potassium pump, a major cellular ATP consumer required for maintenance of the cellular membrane potential [93,94]. Thus, disruption of magnesium or other essential metal ion binding by zinc may contribute to dysregulation of metabolism and enhanced ATP demand.

Mitochondrial dysfunction or damage may also enhance ATP demand. During mitochondrial dysfunction, ATP synthase may consume ATP in order to generate and maintain mitochondrial membrane potential [37,95]. Our data provides significant evidence for disruption of the mitochondrial membrane potential, including dose-dependent depolarization of the inner membrane and up-regulation of the translocase of the inner membrane subunit 17b (*Timm17b*) in RASMC cells. TIMM17B is an essential component of the TIM23 complex required for protein import into the mitochondrial matrix [96]. Previous studies have indicated that mitochondrial stress induces the expression of mitochondrial import machinery in order to efficiently import proteins required for the mitochondrial unfolded protein response during periods of reduced mitochondrial membrane potential [80]. Therefore, up-regulation of *Timm17b* may indicate a mechanism to ensure mitochondrial quality control and function during chronic zinc supplementation.

## Conclusions

In summary, the differential effects of zinc on rat vascular endothelial and smooth muscle cells observed *in vivo* [15], [16], [17] are concomitant with differential effects on mitochondria and energy metabolism *in vitro*. We observed cell-type specific changes in gene expression related to several mitochondrial genes as well as corresponding changes in mitochondrial form and function. These differences may arise from cell-type specific differences in zinc-buffer and storage capacity requiring different mechanisms for the maintenance of mitochondrial quality control and function with chronic zinc supplementation. This study examines the effect of zinc supplementation over 7 days in order to represent the long-term effects of zinc exposure during stent application *in vivo*. The identification of mitochondria as an important target of zinc that is differently affected in the vascular endothelial and smooth muscle cells is important for better understanding how zinc-based implants affect tissue homeostasis *in vivo*.

## Declaration of Competing Interest

The authors declare that they have no known competing financial interests or personal relationships that could have appeared to influence the work reported in this paper.

## Appendix Supplementary materials

Image, application 1
