# Supplementary material for: Bioabsorbable metal zinc differentially affects mitochondria in vascular endothelial and smooth muscle cells
Source: Biomater Biosyst. 2021 Aug 26;4:100027. doi: 10.1016/j.bbiosy.2021.100027 (PMC9934485; doi:10.1016/j.bbiosy.2021.100027)
Supplement: Supplementary file 1 [file mmc1.docx]

**Supplementary Material**

**924336**

**Figure S1.** Cell doubling time and cell viability analysis for (a) RAENDO and (b) RASMC cells following 7 days of treatment with 0-100 µM ZnSO_4_. Cell doubling time was calculated with total cell count at each passage. A Trypan blue (0.04% in PBS) exclusion assay was used to calculate percent viability at Day 7. Significant differences were only observed between Control and 100 µM ZnSO_4_ treatment as indicated by asterisks: * *p*-value ≤ 0.05, ** *p*-value ≤ 0.01, *** *p*- value ≤ 0.001. n =2-3.

**Supplementary Methods**

*Cell growth analysis*

To evaluate change in cellular growth rate, a known number of cells was seeded at each passage (initial seed count) and time elapsed (t) between passages was recorded. Cells were isolated by trypsinization (0.25% trypsin/ EDTA; Sigma, T4049) and resuspended in 1 mL of media or phosphate buffered saline (PBS) (Day 7 only). Triplicate hemocytometer counts were performed to determine final cell count at time t. Doubling time was calculated by the following formula:

$$Doubling time=\frac{t\cdot\ln(2)}{\ln\left( \frac{Final cell count at time t}{Intial seed count} \right)}$$

*Cell viability analysis*

On Day 7, cells were isolated by trypsinization and resuspended in 1 mL PBS. 50 µL of 0.4% Trypan blue in PBS and 50 µL of cell suspension were mixed and incubated at room temperature for approximately 3 minutes. Following incubation, triplicate hemocytometer counts recorded clear (viable) and blue (nonviable) cells. Percent cell viability was determined by the following formula:

$$Cell viability \left( \% \right)=\frac{total number of viable cells}{total number of cells} \times100$$

*Statistics*

Data are presented as means ± standard deviation. A two-way analysis of variance (ANOVA) or mixed-effects analysis with Dunnett’s multiple comparisons post-hoc analyses were performed for each data set. *P* values ≤ 0.05 were considered significant. All statistical analyses were performed using GraphPad Prism version 9.1.0 (San Diego, CA).
